# Association between allopurinol and mortality among Japanese hemodialysis patients: results from the DOPPS

**DOI:** 10.1007/s11255-014-0731-0

**Published:** 2014-06-08

**Authors:** Yuki Tsuruta, Kosaku Nitta, Tadao Akizawa, Shunichi Fukuhara, Akira Saito, Angelo Karaboyas, Yun Li, Friedrich K. Port, Bruce M. Robinson, Ronald L. Pisoni, Takashi Akiba

**Affiliations:** 1 Department of Medicine, Kidney Center, Tokyo Women’s Medical University, 8-1 Kawadacho, Shinjuku-ku, Tokyo, 1628666 Japan; 2 Showa University School of Medicine, Shinagawa, Tokyo, Japan; 3 Department of Epidemiology and Healthcare Research, Graduate School of Medicine and Public Health, Kyoto University, Kyoto, Japan; 4 Yokohama Daiichi Hospital, Tokai University School of Medicine, Yokohama-shi, Kanagawa, Japan; 5 Arbor Research Collaborative for Health, Ann Arbor, MI USA; 6 Department of Biostatistics, University of Michigan School of Public Health, Ann Arbor, MI USA; 7 Department of Blood Purification, Kidney Center, Tokyo Women’s Medical University, Tokyo, Japan

**Keywords:** Allopurinol, Cardiovascular disease, Chronic kidney disease, DOPPS, Hemodialysis, Hyperuricemia

## Abstract

**Purpose:**

Allopurinol, for treating hyperuricemia, is associated with lower mortality among hyperuricemic patients without chronic kidney disease (CKD). Greater allopurinol utilization in hemodialysis (HD) in Japan versus other countries provides an opportunity for understanding allopurinol-related HD outcomes.

**Methods:**

Data from 6,252 Japanese HD patients from phases 1–3 of the Dialysis Outcomes and Practice Patterns Study (1999–2008) at ~60 facilities per phase were analyzed. Mortality was compared for patients prescribed (25 %) versus not-prescribed allopurinol using Cox regression, overall, and in patient subgroups.

**Results:**

Patients prescribed allopurinol were more likely to be younger, male, and non-diabetic, and had higher serum creatinine and lower (treated) serum uric acid levels (mean = 7.0 vs. 8.0 mg/dL, *p* < 0.001). The inverse association between allopurinol prescription and mortality in unadjusted analyses (HR 0.65, 95 % CI 0.52–0.81) was attenuated by covariate adjustment (HR 0.84, 0.66–1.06). In subgroup analyses, allopurinol was associated with lower mortality among patients with no history of cardiovascular disease (CVD) (HR 0.48, 0.28–0.83), but not among patients with CVD (HR 1.00, 0.76–1.32). A similar pattern was seen outside Japan and for cardiovascular (CV)-related mortality.

**Conclusions:**

Allopurinol prescription was not significantly associated with case-mix-adjusted mortality in Japanese HD patients overall, but was associated with lower all-cause and CV-related mortality in the subgroup of patients with no prior CVD history. These findings in HD patients may be related to findings in non-dialysis CKD patients showing lower CV event rates and mortality, and improved endothelial function with allopurinol prescription. These results are useful for designing future trials of allopurinol use in HD patients.

## Introduction

Hyperuricemia has been consistently associated with cardiovascular disease (CVD) [1] and with other CVD risk factors, such as hypertension, chronic kidney disease (CKD), and metabolic syndrome [2–4]. Furthermore, hyperuricemia is a risk factor for acute kidney injury in patients undergoing cardiovascular surgery such that when uric acid levels were decreased using rasburicase prior to cardiovascular (CV) surgery in patients with GFR <45 mL/min/1.73 m^2^, subsequent risk of acute kidney injury and markers of renal tubular injury were significantly lower versus hyperuricemic patients not receiving rasburicase [5]. Whether uric acid level is an independent risk factor for CVD is still debated. The Framingham heart study indicated that uric acid does not have a causal role in the development of coronary heart disease, death from CVD, or death from all causes [6]. However, a meta-analysis of 26 eligible studies suggested that hyperuricemia may marginally increase the risk of coronary heart disease events, independently of traditional coronary heart disease risk factors [7], and a large-scale epidemiologic study of males demonstrated that patients with gout had a higher risk of CV mortality [8]. Furthermore, a recent Chinese general population study (*N* = 90,393) showed hyperuricemia as an independent risk factor for all-cause mortality (HR 1.16) and CV-related mortality (HR 1.39) [9]. Elevated serum uric acid levels are common in hemodialysis (HD) patients [10–12], and CVD represents a common risk of morbidity and mortality in HD patients [13]. Hyperuricemia has been shown to be an independent risk factor of CVD mortality in HD patients with gout [10–14]. There is little information on survival benefit from lowering uric acid in dialysis patients, although a lower risk of all-cause mortality was found with allopurinol therapy in hyperuricemic patients without CKD (*N* = 9,924) after adjustment for many prognostic factors (HR 0.77) [15].

The survival benefit of targeting lower levels of uric acid in HD patients has not been reported yet. Allopurinol is rarely used in the Dialysis Outcomes and Practice Patterns Study (DOPPS) countries outside of Japan. Therefore, we used the prospective Japanese DOPPS (1999–2008) to examine the relationship of uric acid levels and allopurinol treatment with survival in HD patients.

## Methods

### Patients

The present study includes data from 6,252 HD patients from the Japan DOPPS (JDOPPS): 2,461 patients from 64 facilities in DOPPS I (1999–2001), 1,883 patients from 57 facilities in DOPPS II (2002–2004), and 1,908 patients from 62 facilities in DOPPS III (2005–2008). The DOPPS design has been previously described [16, 17]. Patients with missing uric acid data (4 %) or with cancer (7 %) were excluded. Allopurinol prescription was defined as having a prescription for allopurinol at study baseline. Adherence to allopurinol prescription was not determined. Baseline data on demographics, laboratory data (measured pre-dialysis), comorbid conditions, and medications were collected at study entry. However, in DOPPS I, uric acid and allopurinol prescription data were from 4 months after study entry which was when these data were first collected; other patient characteristics were also updated with data from 4 months after study entry. Kt/V was calculated according to the second-generation formula of Daugirdas [18].

### Statistical analysis

Linear mixed models for continuous variables and generalized estimating equations (GEE) models with logit link function for binary variables were used to determine associations with allopurinol prescription. Models accounted for facility clustering effects and were adjusted for DOPPS phase. Linear mixed models with systolic blood pressure (SBP) as the dependent variable were used to assess the association between allopurinol, uric acid, and SBP. Model adjustments were consistent with model adjustments in Fig. 2, Model 5 (but not adjusted for history of hypertension). Among the 65–70 % of patients with baseline C-reactive protein (CRP) measured in phases 2–3 (*N* = 2,585), adjusted GEE models were used to assess the association between allopurinol and high CRP (≥3 mg/L).

For mortality analyses, time-at-risk began at study entry for DOPPS II and III patients, and 4 months after study entry for DOPPS I patients. Participants were followed until the earliest of death, or 7 days following departure from the facility for switch to home-based dialysis, withdrawal from dialysis, return of renal function, kidney transplantation, transfer to another facility, or study end. Mean follow-up time was 20.9 months (maximum 37.1 months). Cox models stratified by study phase and adjusted for expanding sets of patient characteristics were used to assess the relationship of all-cause mortality with either uric acid levels or allopurinol prescription. Since normal range for uric acid differs between males and females [19], interaction between uric acid and gender was assessed. The proportional hazards assumption for Cox regression was confirmed in each phase for model covariates using log(time) interaction terms and log–log survival plots. Deaths due to any of the following reasons were considered CV related: (1) myocardial infarction, acute, (2) atherosclerotic heart disease, (3) cardiomyopathy, (4) cardiac arrhythmia, (5) cardiac arrest, causes unknown, and (6) congestive heart failure.

Propensity scores were calculated using covariates listed in Fig. 2 (Model 5). Scores were divided into quintiles (range 4–81 %, median = 25 %) and adjusted for in a Cox model with observed allopurinol prescription as the variable of interest.

Differences in the association of allopurinol with all-cause mortality in patient subgroups were assessed by testing the interaction between allopurinol and several indicators: gender, age, vintage, albumin, and the eight comorbidities listed in Table 1 with >5 % prevalence. Each interaction effect was tested in a separate model and adjusted as in previous analyses. In a post hoc analysis of possible effect modification by CVD, patients were divided into two groups: those with at least one of four CV-related comorbidities [coronary artery disease (CAD), congestive heart failure (CHF), peripheral vascular disease (PVD), other CVD], labeled CV4 = 1, and those with none of these CV comorbidities.

**Table 1:** Characteristics of Japanese HD patients by allopurinol use

Patient characteristics (mean ± SD or %)	All patients (*N* = 6,252)	With allopurinol use (*N* = 1,561)	No allopurinol use (*N* = 4,691)	*p* value*
Uric acid (mg/dL)	7.7 ± 1.5	7.0 ± 1.5	8.0 ± 1.4	<0.001
Uric acid >9 mg/dL	17 %	8 %	20 %	<0.001
*Demographics*				
Age (years)	60.8 ± 12.8	59.7 ± 12.2	61.2 ± 13.0	<0.001
Male (%)	61 %	68 %	59 %	<0.001
Duration on dialysis (months)	77.1 ± 81.0	79.5 ± 79.3	76.3 ± 81.5	0.09
Dry weight (kg)	52.8 ± 10.6	54.9 ± 11.1	52.1 ± 10.3	<0.001
*Laboratory data*				
Serum albumin (g/dL)	3.77 ± 0.44	3.79 ± 0.41	3.77 ± 0.45	<0.001
Serum creatinine (mg/dL)	10.7 ± 3.0	11.3 ± 3.2	10.5 ± 3.0	<0.001
nPCR	1.03 ± 0.23	1.08 ± 0.24	1.02 ± 0.23	<0.001
Cachectic (%)	5 %	3 %	5 %	0.001
WBC count (1,000 per mm^3^)	6.0 ± 2.0	6.1 ± 2.0	6.0 ± 2.0	0.04
Hemoglobin (g/dL)	9.9 ± 1.4	10.0 ± 1.4	9.9 ± 1.4	0.002
Serum calcium_alb_ (mg/dL)	9.3 ± 0.9	9.4 ± 0.9	9.2 ± 1.0	<0.001
Serum phosphorus (mg/dL)	5.6 ± 1.6	5.8 ± 1.6	5.6 ± 1.6	<0.001
Single pool (Kt/V)	1.31 ± 0.30	1.30 ± 0.29	1.31 ± 0.30	0.7
*Comorbid conditions*				
Coronary artery disease	28 %	26 %	28 %	0.01
Other cardiovascular disease	28 %	28 %	28 %	0.6
Cerebrovascular disease	14 %	14 %	14 %	0.2
Congestive heart failure	18 %	16 %	18 %	0.04
Diabetes mellitus	31 %	25 %	33 %	<0.001
GI bleeding	4 %	4 %	4 %	0.5
Hypertension	67 %	67 %	67 %	0.2
Lung disease	2 %	2 %	2 %	0.5
Neurological disease	7 %	4 %	7 %	<0.001
Psychiatric disorder	3 %	3 %	3 %	0.2
Peripheral vascular disease	13 %	11 %	14 %	<0.001
Recurrent cellulitis	3 %	2 %	3 %	0.01

* *p* value represents association of allopurinol with each patient characteristic, adjusted for DOPPS phase and accounting for facility clustering effects. Linear mixed models for continuous variables and generalized estimating equations (GEE) models with logit link function for binary variables were used

To reduce the impact of unmeasured patient-level confounders in mortality analyses of allopurinol prescription, patient survival was also examined in an instrumental variable (IV) approach. Implemented separately for patients with CV4 and without CV4, the IV method used the two-stage residual inclusion (2SRI) [20] methodology with dialysis facilities as the instruments to examine mortality, adjusting for variables used in Model 5 in Fig. 2.

To supplement this analysis of Japanese patients, adjusted Cox models were used to investigate allopurinol and mortality in 15,639 patients from all 11 other DOPPS countries (Australia, Belgium, Canada, France, Germany, Italy, New Zealand, Spain, Sweden, UK, and USA) in phases 2 and 3.

For missing covariate data, we used the sequential regression multiple imputation method implemented by IVEware [21] and analyzed using the MIAnalyze procedure in SAS 9.2. Among all imputed variables, normalized protein catabolic rate (nPCR) (18 %) and single pool Kt/V (17 %) had the highest rates of missingness. All statistical analyses were performed using the SAS statistical package, version 9.2 (SAS Institute, Cary, NC).

## Results

### Serum uric acid level, allopurinol prescription, and HD patient characteristics

Among the 6,252 Japanese HD patients included in the analysis, the mean age was 60.8 years with a mean of 77.1 months of prior maintenance dialysis (Table 1). Patient uric acid levels showed a Gaussian distribution. The mean ± SD serum uric acid concentration was 7.7 ± 1.5 mg/dL (7.0 ± 1.5 vs. 8.0 ± 1.4 mg/dL) in patients prescribed versus not-prescribed allopurinol, respectively, (*p* < 0.001) and varied substantially across Japanese dialysis units. Facilities that prescribed allopurinol to a higher percentage of patients had lower average uric acid levels (0.16 mg/dL lower uric acid level for every 10 % higher facility percentage allopurinol prescription, *p* < 0.001, Table 2).

**Table 2:** Characteristics of Japanese HD patients by quartile of facility  % allopurinol use

Patient characteristics (mean ± SD OR %)	Q1: 0–9 % use (*N* = 1,578)	Q2: 9–22 % use (*N* = 1,557)	Q3: 22–34 % use (*N* = 1,524)	Q4: 34–100 % use (*N* = 1,593)	*p* value*
Uric acid (mg/dL)	8.0 ± 1.4	7.8 ± 1.4	7.7 ± 1.5	7.3 ± 1.4	<0.001
Uric acid >9 mg/dL	22 %	19 %	17 %	10 %	<0.001
*Demographics*					
Age (years)	60.4 ± 13.2	61.1 ± 12.7	60.9 ± 13.1	61.0 ± 12.4	0.4
Male (%)	60 %	62 %	61 %	62 %	0.6
Duration on dialysis (months)	87.2 ± 86.9	68.4 ± 76.8	73.2 ± 79.5	79.3 ± 78.9	0.9
Dry weight (kg)	52.3 ± 10.9	53.2 ± 10.5	52.7 ± 10.3	53.1 ± 10.7	0.1
*Laboratory data*					
Serum albumin (g/dL)	3.78 ± 0.43	3.79 ± 0.47	3.75 ± 0.46	3.76 ± 0.39	0.6
Serum creatinine (mg/dL)	10.7 ± 3.0	10.6 ± 3.0	10.6 ± 3.1	10.9 ± 3.1	0.3
nPCR	1.02 ± 0.22	1.02 ± 0.23	1.03 ± 0.23	1.06 ± 0.24	0.08
Cachectic (%)	5 %	5 %	4 %	4 %	0.4
WBC count (1,000 per mm^3^)	6.0 ± 2.0	6.1 ± 2.0	6.0 ± 2.0	6.0 ± 2.0	0.6
Hemoglobin (g/dL)	10.1 ± 1.4	9.9 ± 1.3	9.8 ± 1.5	10.0 ± 1.5	0.5
Serum calcium_alb_ (mg/dL)	9.2 ± 1.0	9.2 ± 0.9	9.3 ± 1.0	9.3 ± 0.9	0.007
Serum phosphorus (mg/dL)	5.6 ± 1.5	5.5 ± 1.5	5.6 ± 1.6	5.8 ± 1.7	0.09
Single pool (Kt/V)	1.35 ± 0.30	1.29 ± 0.28	1.30 ± 0.32	1.30 ± 0.28	0.2
*Comorbid conditions*					
Coronary artery disease	25 %	30 %	26 %	29 %	0.2
Other cardiovascular disease	26 %	30 %	27 %	29 %	0.5
Cerebrovascular disease	11 %	14 %	14 %	17 %	0.05
Congestive heart failure	13 %	23 %	16 %	18 %	0.3
Diabetes mellitus	27 %	36 %	31 %	32 %	0.5
GI bleeding	3 %	4 %	3 %	5 %	0.3
Hypertension	63 %	69 %	66 %	70 %	0.02
Lung disease	1 %	2 %	2 %	3 %	0.06
Neurological disease	5 %	7 %	7 %	7 %	0.2
Psychiatric disorder	2 %	4 %	4 %	4 %	0.05
Peripheral vascular disease	11 %	15 %	13 %	14 %	0.1
Recurrent cellulitis	2 %	4 %	4 %	3 %	0.8

* *p* value represents association of facility % of patients prescribed allopurinol (continuous variable) with each patient characteristic, adjusted for DOPPS phase and accounting for facility clustering effects. Linear mixed models for continuous variables and generalized estimating equations (GEE) models with logit link function for binary variables were used

Allopurinol prescription rate was 25 % in the study sample and varied greatly across JDOPPS facilities (range 0–100 % of facility patients; median = 22 %, interquartile range 9–33 %). A trend (*p* = 0.06) toward decreased allopurinol prescription was seen from JDOPPS 1–3: 28, 26, and 21 %, respectively.

Table 1 shows baseline characteristics for patients prescribed versus not-prescribed allopurinol. On average, patients prescribed allopurinol were younger, more likely male, and had higher mean dry weight and a lower prevalence of several comorbidities. In addition, patients prescribed allopurinol displayed higher levels for serum albumin, creatinine, nPCR, white blood cell count, hemoglobin, albumin-corrected serum calcium, and serum phosphorus. Despite allopurinol prescription being positively associated with many indicators of better health status, there was no strong association between higher facility percentage allopurinol prescription and indicators of better health status (Table 2).

In adjusted models, allopurinol prescription was associated with slightly lower SBP (−1.3 mmHg; 95 % CI −2.8, +0.1), but greater facility percentage allopurinol prescription was not associated with SBP (*p* = 0.8). In addition, uric acid was weakly associated with SBP (−0.2 mmHg; 95 % CI −0.6, +0.2 per 1 mg/dL higher uric acid). High baseline CRP was defined as CRP ≥3 mg/L and present in 33 % of patients. For patients prescribed versus not-prescribed allopurinol, the adjusted odds ratio of having a CRP ≥3 mg/L was 0.92 (95 % CI 0.75–1.14) when using adjustment as in Fig. 2, Model 5.

### Serum uric acid level and all-cause mortality

In unadjusted analyses, a higher uric acid level (per 1 mg/dL) was associated with lower mortality (HR 0.86, 95 % CI 0.80–0.92, Table 3, Model 1). However, the association of serum uric acid with mortality was attenuated to a HR 0.95 (95 % CI 0.89–1.01) (Table 3, Model 5) after adjustment for patient demographics, comorbidity, and nutritional status, and to HR 0.92 (95 % CI 0.86–0.99) with additional adjustment for allopurinol prescription (Table 3, Model 6). Mortality analysis by quintile of uric acid level also showed progressively lower mortality with higher uric acid concentration, consistent with the results treating uric acid as a continuous variable (Table 3). There was no evidence of interaction between uric acid and gender (*p* for interaction = 0.8).

**Table 3:** Relationship of mortality with uric acid, as a continuous variable and by uric acid quintile, for various levels of adjustment

*N* patients = 6,252, *N* deaths = 516	Model 1	Model 2	Model 3	Model 4	Model 5	Model 6
Per 1 mg/dL uric acid	0.86 (0.80–0.92)	0.90 (0.84–0.96)	0.92 (0.86–0.98)	0.96 (0.90–1.02)	0.95 (0.89–1.01)	0.92 (0.86–0.99)
*Quintile Model*						
2.1–6.5 mg/dL	1.00 (ref.)	1.00 (ref.)	1.00 (ref.)	1.00 (ref.)	1.00 (ref.)	1.00 (ref.)
6.6–7.2 mg/dL	0.82 (0.61–1.10)	0.88 (0.66–1.16)	0.88 (0.66–1.18)	1.02 (0.77–1.36)	0.99 (0.75–1.32)	0.94 (0.70–1.27)
7.3–8.0 mg/dL	0.70 (0.54–0.93)	0.77 (0.60–1.01)	0.85 (0.65–1.10)	0.98 (0.74–1.29)	0.96 (0.73–1.26)	0.89 (0.67–1.19)
8.1–8.8 mg/dL	0.60 (0.45–0.80)	0.72 (0.54–0.96)	0.79 (0.60–1.06)	0.96 (0.71–1.29)	0.93 (0.68–1.25)	0.84 (0.61–1.16)
8.9–16.4 mg/dL	0.57 (0.43–0.76)	0.67 (0.51–0.89)	0.70 (0.53–0.93)	0.86 (0.64–1.16)	0.82 (0.61–1.10)	0.74 (0.55–1.02)

Cox model hazard ratios (95 % confidence interval) of all-cause mortality

Model 1: stratified by phase, and accounting for facility clustering effects

Model 2: Model 1 + adjusted for age, gender, and years on dialysis

Model 3: Model 2 + 12 comorbidities listed in Table 1

Model 4: Model 3 + serum albumin, creatinine, dry weight, nPCR, cachexia, white blood cell count

Model 5: Model 4 + hemoglobin, calcium*phosphorus product, single pool Kt/V

Model 6: Model 5 + allopurinol use

### Allopurinol prescription and mortality

**Fig. 1:**
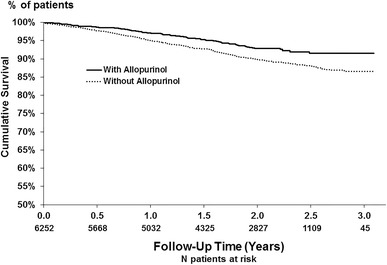
Kaplan–Meier survival plot for Japanese HD patients prescribed versus not-prescribed allopurinol. The number of deaths/total patients among patients not-prescribed versus prescribed allopurinol was 421/4691 and 95/1561, respectively. Log-rank test *p* < 0.001

**Fig. 2:**
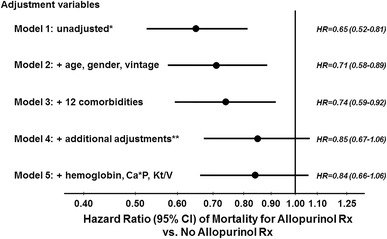
Allopurinol and all-cause mortality, by levels of adjustment. *Unadjusted Cox model stratified by DOPPS phase and accounted for facility clustering; **additional adjustments: serum albumin, creatinine, dry weight, nPCR, cachexia, and white blood cell count. Adjusting for baseline SBP rather than for history of hypertension gave similar results in a sensitivity analysis

The mortality rate during study follow-up was 4.7 per 100 patient years overall, and was 3.4 and 5.2 in patients prescribed versus not-prescribed allopurinol, respectively. A Kaplan–Meier plot (Fig. 1) indicated a consistent survival advantage throughout the study follow-up for patients prescribed allopurinol (log-rank *p* < 0.001). The results of all-cause mortality Cox models adjusted for expanding sets of patient characteristics are shown in Fig. 2. The survival advantage for patients prescribed versus not-prescribed allopurinol was considerably attenuated from HR 0.65 (95 % CI 0.52–0.81) in unadjusted analyses to HR 0.84 (95 % CI 0.66–1.06) after adjustment for demographics, laboratory data, and comorbid conditions. Models adjusted for quintiles of the patient’s propensity to be prescribed allopurinol yielded results similar to the fully adjusted Cox model: HR 0.85 (95 % CI 0.68–1.06). Testing for effect modification failed to show a significant difference across propensity score quintiles (*p* for interaction = 0.5). A HR 0.92 (95 % CI 0.62–1.38) was observed for CV-related mortality in a Cox model adjusted as in Fig. 2, Model 5.

**Fig. 3:**
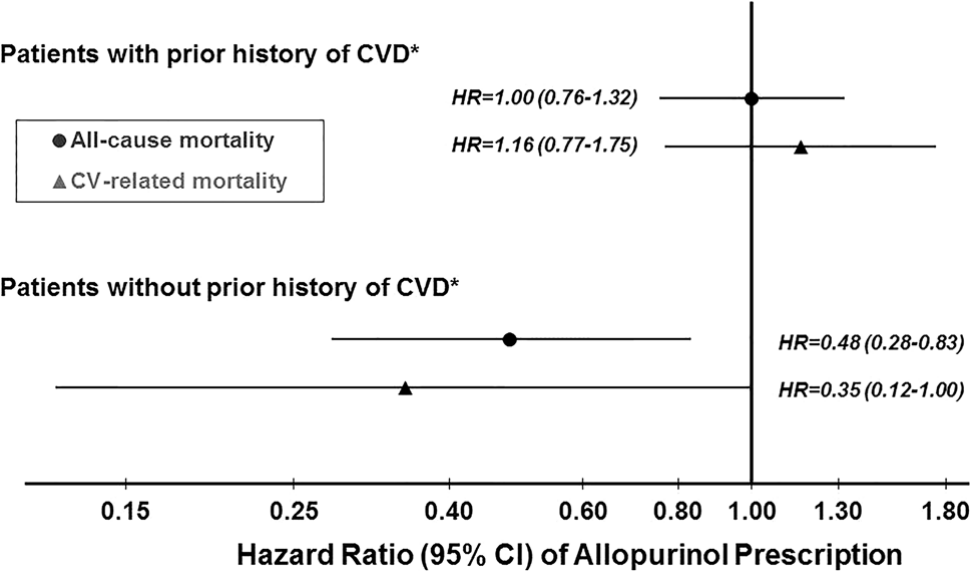
Allopurinol and all-cause mortality: interaction with CV4. *CV4 defined as prior history of at least one of four cardiovascular-related comorbidities (coronary artery disease, congestive heart failure, peripheral vascular disease, or other cardiovascular disease). Other CVD includes cardiac arrest, chronic atrial fibrillation, paroxysmal/recurrent atrial fibrillation, other arrhythmias, permanent pacemaker implanted, automatic implanted cardiac defibrillator (AICD), pericarditis, valvular heart disease by echo/cath, and prosthetic heart valve. The two Cox models, one each for all-cause and CV-related mortality, were adjusted as in Fig. 2, Model 5, except for the four CV4 comorbidities. *P* value for interaction of allopurinol*CV4 indicator was 0.02 for all-cause mortality and 0.03 for CV-related mortality. *N* = 3,239 patients (389 deaths, 146 CV related) with prior history of CVD and *N* = 3,013 patients with no prior history of CVD (127 deaths, 38 CV related)

Subgroup analyses (data not shown) showed a stronger association of allopurinol with lower mortality among younger patients, patients without neurological disease, and patients without CV-related disease. In a post hoc analysis, 48 % of patients did not have any of the four CV-related comorbidities of CAD, CHF, PVD, or other CVD (no-CV4). The adjusted HR of all-cause mortality for patients prescribed versus not-prescribed allopurinol was significantly stronger (*p* for interaction = 0.02) among patients without CV4 (HR 0.48, 95 % CI 0.28–0.83) than among patients with CV4 (HR 1.00, 95 % CI 0.76–1.32) (Fig. 3). Similarly, the association of allopurinol with CV-related mortality was stronger among patients without versus with CV4 (Fig. 3; *p* for interaction = 0.03). In the remaining 11 DOPPS countries, allopurinol was associated with a trend toward lower adjusted all-cause mortality in the no-CV4 subgroup (HR 0.82, 95 % CI 0.59–1.14). However, this international analysis was limited in that only 11 % of patients were prescribed allopurinol and only 24 % of patients did not have any of the CV4 comorbidities.

Two separate IV analyses, which help account for unmeasured patient-level confounding, were conducted for each CV4 subgroup of patients were directionally consistent with the above findings. A trend again suggested lower mortality for patients prescribed allopurinol among the no-CV4 subgroup (HR 0.56, 95 % CI 0.24–1.32). In contrast, there was no indication for lower mortality with allopurinol prescription among patients in the CV4 group (HR 1.41, 95 % CI 0.81–2.46).

## Discussion

Few studies have related allopurinol use with outcomes in HD patients and have typically had small cohorts. The current study of HD patients in Japan, where mortality rates are much lower than in Europe or North America [22], is by far the largest cohort study of allopurinol prescription and outcomes in HD patients. The large sample size and detailed multicenter DOPPS data along with the relatively high use of allopurinol in Japanese HD patients (twofold higher than in most other DOPPS countries) have provided this opportunity to understand additional aspects of allopurinol’s use and effectiveness in HD patients, not studied previously. The relatively high use of allopurinol among HD patients in Japan can be ascribed to the importance Japanese nephrologists place upon treating hyperuricemia, particularly for reducing CVD-related mortality, as noted in a recent survey of 595 Japanese nephrologists by Nakaya et al. [23].

In this current study, Japanese HD patients prescribed allopurinol (versus not) were younger, more likely to be male, had lower comorbidity burden, and displayed laboratory values suggestive of better health status overall. After adjustment for numerous measures of patient health status and baseline laboratory measures, patients prescribed allopurinol had a ~15 % lower mortality rate compared with patients not-prescribed allopurinol, but the association was not statistically significant (*p* = 0.1). However, a post hoc patient subgroup analysis demonstrated a consistent pattern of lower mortality rates when allopurinol was prescribed to patients with no prior history of several CV-related comorbidities. Conversely, no appreciable survival benefit was seen when allopurinol was prescribed to patients who had a prior history of these CV-related comorbidities (Fig. 3). This pattern of allopurinol benefit for particular patient subgroups was further supported by a similar finding in the international DOPPS data exclusive of Japan and by IV analyses in Japan. Additionally, the association of allopurinol with substantially lower CV-related mortality in Japanese HD patients without any of the four CV-related comorbidities lends further support for a possible effect of allopurinol involving CV-related processes.

This observation of possible greater benefit of allopurinol in relatively healthy patients without a prior history of CVD suggests that allopurinol may help protect against the progression or onset of CVD in HD patients who are yet relatively free of advanced CVD. Thus, allopurinol may exert a potential beneficial effect at a key step(s) in the early stages of CVD progression. This hypothesis is consistent with numerous studies in CKD patients and animal studies showing allopurinol to decrease endothelial dysfunction and oxidative stress, with end-stage renal disease patients known to have elevated levels of oxidative stress [3, 24, 25]. Allopurinol is known to reduce serum uric acid levels by xanthine oxidase (XO) inhibition. Many clinical studies have shown that XO inhibition improves endothelial function in patients with chronic heart failure, CKD, diabetes mellitus, and hypertension [3, 26–29]. Baldus et al. [30] have shown that oxipurinol inhibition of XO improved endothelial function independently of changes in uric acid levels in patients with coronary artery disease. Furthermore, in two randomized, placebo-controlled double-blind crossover studies of allopurinol therapy in chronic heart failure patients, George et al. [31] showed a steep dose–response relationship between allopurinol and improved endothelial function ascribed largely to allopurinol’s ability to reduce vascular oxidative stress, and not due to its ability to reduce uric acid levels. In addition, a recent 4-month prospective randomized trial showed that allopurinol therapy improved endothelial dysfunction in hyperuricemic individuals having normal renal function, with allopurinol resulting in decreased serum uric acid and SBP levels, while increasing flow-mediated dilatation and eGFR levels compared with baseline. In contrast, no change in flow-mediated dilatation was seen in control hyperuricemic patients not receiving allopurinol [32]. Moreover, it has been reported recently that allopurinol also reduced left ventricular mass index and improved flow-mediated dilatation among CKD stage 3 patients [33]. In another prospective, randomized trial, Goicoechea et al. [34] found that CKD patients with eGFR <60 mL/min/1.73 m^2^ randomized to allopurinol treatment for up to 24 months, displayed a 71 % lower relative risk of CV events, a 62 % lower relative risk of hospitalization, lower uric acid and CRP levels, and a much lower rate of renal disease progression compared with standard therapy. Thus, there is an expanding body of evidence pointing to possible therapeutic effects of allopurinol for improving endothelial function independent of reducing uric acid levels.

A number of clinical trials have shown reductions in SBP and CRP levels in hyperuricemic CKD patients treated with allopurinol over a relatively short time period [3]. Kanbay et al. showed that allopurinol therapy of hyperuricemic patients with GFR >60 mL/min/1.73 m^2^ resulted in reductions in systolic and diastolic blood pressure, and CRP levels while raising GFR during 3 months of therapy [35]. In the present study of Japanese patients as treated during routine care, we observed a slightly lower SBP for patients prescribed allopurinol, and with little difference seen in CRP levels for patients prescribed versus not-prescribed allopurinol. Our study results may be expected to differ from those in a clinical trial of allopurinol due to the variations in time on drug and in use of anti-hypertensive agents and other practices across DOPPS facilities, which may impact SBP as well as levels of inflammatory markers.

Randomized clinical trials of allopurinol have been very limited in the HD patient population. Recently, Badve et al. [36] highlighted the challenges in conducting a trial of urate-lowering drugs in CKD patients, and key issues for development of a future large multicenter trial. An important contribution of the current study is that our findings, in particular the effect sizes in different patient subpopulations, serve as uniquely valuable information for the design of future randomized trials regarding allopurinol therapy. An important strength of the DOPPS study is the nationally representative sample of facilities that provide study data in each country.

The present study also found lower mortality rates for Japanese patients with higher uric acid levels similar to what the DOPPS recently reported in an international study of uric acid levels and mortality [37]. A key difference between the previous DOPPS analysis and this current analysis is that the main focus of the previous international paper was uric acid and mortality; allopurinol and mortality were not analyzed because the prescription rate of allopurinol was very low across most DOPPS countries. Our study takes advantage of greater patient- and facility-level variation in allopurinol prescription in Japan where the drug is more commonly prescribed. In contrast, a recent single-center study of 53 HD patients by Antunovic et al. found higher mortality risk for patients having higher uric acid levels [38].

The findings from the present work are subject to methodological limitations. Because the study is observational, it does not allow conclusions regarding cause and effect relationships between allopurinol prescription and outcomes. The indication for allopurinol may have been for clinical gout in some patients; unfortunately, the reason for the prescription was not collected. Since generally healthier patients, on the basis of age, diabetes, creatinine, and other covariates, were prescribed allopurinol (Table 1), an instrumental variable approach was implemented to reduce the influence of unmeasured patient-level confounders. However, we cannot rule out the possibility of remaining residual confounding. The authors acknowledge that the subgroup analyses stratified by prior CVD were conducted only after observing a strong interaction effect between allopurinol and various CVD-related comorbidities; because this interaction was not considered a priori, the results can only be viewed as hypothesis generating. Finally, allopurinol prescription was based on baseline data, whereas time on drug, adherence to medication use, and allopurinol prescription during follow-up were not assessed.

In conclusion, we have provided a detailed investigation of the relationship between allopurinol prescription and mortality in HD patients. The large proportion of patients prescribed allopurinol in Japan has provided a unique opportunity for this investigation. After accounting for differences in patient demographics, comorbid conditions, and numerous laboratory measures, we observed a lower but not statistically significant association between allopurinol prescription and mortality overall. Though no association was found between allopurinol and mortality in patients with a prior history of CVD, we have observed a substantial survival benefit for allopurinol prescription among patients with no prior history of CV-related comorbidities. The reasons for this dichotomy are not known at the present time but possibly indicate that allopurinol may help protect against the progression or onset of CVD in HD patients who are yet relatively free of advanced CVD. Our results from this large multicenter cohort study of allopurinol prescription can serve as an important source of information for the design of future randomized controlled trials of allopurinol use in HD patients.
